# Exploring the association between caffeine intake and benign prostatic hyperplasia: results from the NHANES 2005–2008

**DOI:** 10.3389/fnut.2024.1511607

**Published:** 2025-01-13

**Authors:** Fei Zhang, Andong Zhang, Junyue Tao, Meng Zhang, Chaozhao Liang

**Affiliations:** ^1^Department of Urology, The First Affiliated Hospital of Anhui Medical University, Hefei, China; ^2^Institute of Urology, Anhui Medical University, Hefei, China; ^3^Anhui Province Key Laboratory of Urological and Andrological Diseases Research and Medical Transformation, Anhui Medical University, Hefei, China

**Keywords:** caffeine intake, coffee, benign prostatic hyperplasia, NHANES, men’s health

## Abstract

**Background:**

Coffee is a physiologically active food component prevalent throughout the world, but the association between caffeine intake and benign prostatic hyperplasia (BPH) has been limited in extensive epidemiological studies.

**Methods:**

We conducted a cross-sectional study to evaluate the association between caffeine intake and BPH in adults in the United States using data from the National Health and Nutrition Examination Survey (NHANES) 2005–2008. Caffeine intake (mg/day) was evaluated based on a 24-h dietary recall. Multivariate logistic regression was used to analyze the independent relationship between caffeine intake and BPH, and the results are presented as odds ratio (OR) and 95% confidence interval (CI), subgroup analysis was also performed.

**Results:**

A total of 2,374 participants were analyzed. After fully adjusting for potential confounders, logistic regression analysis revealed that higher caffeine intake was associated with a greater risk of BPH (ORT3vs1 = 1.52, 95% CI: 1.01–2.27; *p* = 0.04). In addition, this relationship was consistently observed across different subgroups, including individuals with lower education levels, a poverty income ratio (PIR) of 1.5 to 3.5, former smokers, married/living with partner individuals, those with uric acid levels of 5.5 to 6.5 mg/dL, those with hypertension, and those without cardiovascular disease (CVD).

**Conclusion:**

This study is the first to find a positive correlation between caffeine intake and BPH, but further research is needed to determine the exact causal relationship between these factors.

## 1 Introduction

Benign prostatic hyperplasia (BPH), a disease characterized by uncontrolled overgrowth of epithelial and smooth muscle cells located in the transition zone of the urethra, is one of the most common diagnoses made by urologists (1). BPH mainly manifests as lower urinary tract symptoms (LUTS), and its incidence has been on the rise in recent years. Epidemiological surveys have reported that this disease affects over 15 million men, particularly those over the age of 50, and is a considerable economic burden on society (2). However, the specific mechanism of BPH is unclear, as multifactorial disease, age, androgen and estrogen interactions, obesity, diabetes, hypertension, hypogonadism, unhealthy lifestyle factors such as smoking, and alcohol consumption may be potential risk factors for the development of BPH (3). Among these factors, dietary factors were found to be associated with BPH (4–9).

Currently, coffee is one of the most frequently consumed drinks worldwide, with caffeine being its primary pharmacologically active compound (10). A survey by the National Coffee Association indicated that nearly 64% of American adults consume coffee daily, amounting to approximately 517 million cups each day (11). This widespread consumption has heightened concerns about caffeine’s potential effects on human health. Epidemiological studies have shown that habitual consumption of coffee can prevent and reduce the incidence and prevalence of type 2 diabetes (12), liver disease (13, 14), depression (15), cardiovascular disease (CVD) (16), and cancer (17, 18). However, drinking coffee can also have negative consequences, such as sleep disturbances, anxiety, and adverse pregnancy outcomes (19). The findings from various epidemiological studies on the connection between coffee intake and BPH remain inconclusive. Two earlier case–control studies of men with BPH who underwent surgery showed no significant relationship between coffee intake and BPH (20, 21). A comprehensive, cross-sectional population-based study revealed that increased coffee intake was positively correlated with BPH (22). However, these studies were limited to coffee intake rather than caffeine intake. A previous experimental study suggested that caffeine is associated with BPH and that caffeine may stimulate cell proliferation by increasing androgen signaling in ventral prostate epithelial cells (4). However, it is unclear whether caffeine intake is linked to the development of BPH in the population.

Given this gap in the literature, we conducted a cohort study with data from the 2005–2008 National Health and Nutrition Examination Survey (NHANES) to explore the link between caffeine intake and BPH.

## 2 Materials and methods

### 2.1 Study population

The NHANES is a cross-sectional study, an ongoing program conducted by the National Center for Health Statistics to assess the health and nutritional status of a noninstitutionalized civilian population in the United States, using a complex multistage probability sampling design that has recruited approximately 5,000 nationally representative individual samples each year since 1999. A standardized questionnaire and two successful 24-h dietary recall surveys were used to gather sociodemographic, nutritional, lifestyle, health, income, and medication information from participants, conducted jointly by the U.S. Department of Health and Human Services (DHHS) and the U.S. Department of Agriculture (USDA). All data for this study came from a publicly available database and received ethical approval from the Institutional Review Board of the National Center for Health Statistics Ethics Review Board (NCHS IRB/ERB Protocol No. #2005-2006; Continuation of Protocol No. #2005-06). All participants are informed of the process and purpose of the study, and provided written informed consent consistent with the Public Health Service Act before any data was collected. All methods were carried out in accordance with relevant guidelines and regulations. More information on the NHANES survey design and process has been reported elsewhere (23). Our study was a secondary data analysis, and the National Center for Health Statistics (NCHS) collected the data. All procedures in this study were performed following the criteria of the Declaration of Helsinki.

**Figure 1:**
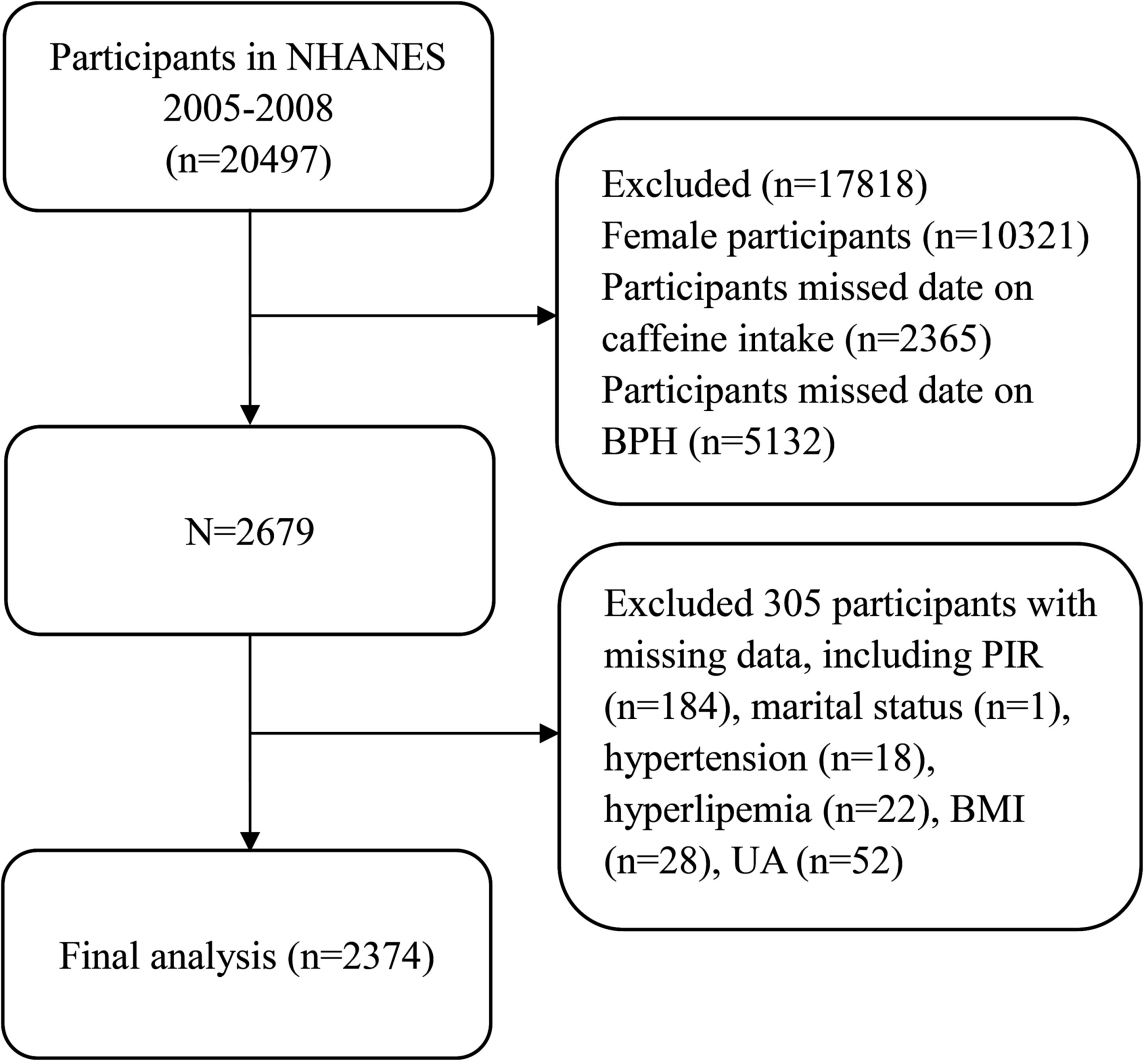
Flow chart of the selection process for participants in the National Health and Nutrition Examination Survey 2005–2008.

This study examined descriptive data over 2 consecutive cycles from 2005–2008, and data, including demographic characteristics, lifestyle habits, physical comorbidities, and other health-related variables, were extracted and aggregated for the final analysis. Participants lacking complete data on caffeine intake or BPH, as well as female subjects, were excluded (*n* = 17,818). In addition, we excluded relevant covariates with missing data, such as body mass index (BMI), uric acid, hypertension, marital status, hyperlipemia, and poverty income ratio (PIR), which also led to the exclusion of participants (*n* = 305); ultimately, a total of 2,374 participants were included for further analysis (Figure 1).

### 2.2 Caffeine intake assessment

Caffeine intake data were collected using a 24-h dietary recall interview, where participants recalled all foods and drinks consumed from midnight to midnight the previous day. Participants underwent two 24-h dietary recall interviews, one at the Mobile Examination Center (MEC) and a second by telephone 3–10 days later (24), with all participants participating in both.

Two reliable 24-h dietary recalls were obtained using the computer-assisted Automated Multiple-Pass Method (AMPM) from the United States Department of Agriculture (USDA). According to the USDA Food and Nutrition Database for Dietary Studies (FNDDS), participants reported caffeine intake from any source (24–26). The mean caffeine intake from the two 24-h recalls was used in the analysis, and daily caffeine intake was divided into three groups according to tertiles (T): T1 (< 106.5 mg/d), T2 (106.5–261.5 mg/d) and T3 (≥261.5 mg/d).

### 2.3 Diagnosis of benign prostatic hyperplasia

Male participants were asked about prostate health: “Have you been diagnosed with an enlarged prostate?” If “yes,” they were asked “Is it benign?” Only those who answered “yes” to both questions were classified as having BPH. Participants with missing data or cancerous enlargement were excluded (27–31).

### 2.4 Covariates

Based on previous studies (27, 32), we selected the following variables as covariates. Sociodemographic factors included age, race (Non-Hispanic White, Non-Hispanic Black, Mexican American, and other races), PIR (< 1.5, 1.5–3.5, and ≥ 3.5), educational level (≥high school, <high school), and marital status (married//living with partner or living alone). Smoking and alcohol consumption were considered health-related lifestyle factors. Nonsmokers were defined as individuals who had smoked fewer than 100 cigarettes in their lifetime, while others were classified as current or former smokers based on their current smoking status. Alcohol consumption was categorized by the amount of drinking into non/light drinkers (1–2 drinks/day) and heavy drinkers (>2 drinks/day).

Clinical variables included BMI (kg/m2) (< 18.5, 18.5 to 25, and ≥ 25), hypertension, hyperlipemia, diabetes, and CVD. Participants who were diagnosed with hypertension, who were currently treated with antihypertensive medications or who had a mean systolic blood pressure ≥ 140 mm/Hg and mean diastolic blood pressure ≥ 90 mm/Hg were defined as having hypertension (33). Uric acid levels were obtained from NHANES laboratory data, and participants’ uric acid values were grouped by tertile (< 5.6 mg/dL, 5.6–6.6 mg/dL, ≥ 6.6 mg/dL). Participants with triglycerides (TG) ≥ 200 mg/dL or who were currently taking lipid-lowering medication were considered to have hyperlipemia. Participants who had self-reported physician-diagnosed diabetes, who were currently taking antidiabetic medication or who had fasting blood glucose >126 mg/dL were considered to have diabetes. Participants were considered to have cardiovascular disease if they had any of the following self-reported conditions: angina pectoris, heart attack, congestive heart failure, coronary artery disease, or stroke (34).

### 2.5 Statistical analysis

To account for NHANES’ complex sampling design, we applied the appropriate sample weights provided by the NHANES. The included variables were tested by multicollinearity, and Variance Inflation Factor (VIF) was used to evaluate them. We expressed continuous variables as mean ± standard deviation, and categorical variables as weighted frequencies (%) and unweighted sample sizes. Continuous variables were compared using independent sample t tests, while categorical variables were compared using chi-square tests. The association between caffeine intake and BPH was analyzed using weighted logistic regression. Three different logistic regression models were used: Model 1 was unadjusted, Model 2 was adjusted for age, race, educational level, PIR, marital status, smoking status, alcohol consumption, and BMI. Model 3 was adjusted for all potential confounding factors, with additional adjustments for diabetes, uric acid, hypertension, hyperlipemia, and CVD in addition to those made in Model 2. The associations between caffeine intake and BPH were analyzed in subgroups using a weighted logistic regression model stratified by educational level, PIR, smoking status, marital status, uric acid, hypertension, and CVD. All analyses were performed with R version 3.6.1, and a *p*-value of less than 0.05 was considered statistically significant.

## 3 Results

### 3.1 Participants characteristics

A total of 2,374 participants were included in our analysis, including 443 BPH participants and 1931 controls. The mean age in the BPH group was 64.7 years, compared with 43.9 years in the control group, and all participants were over 40 years old. Compared to the control group, the BPH group tended to be older and had a greater proportion of former smokers and those with a history of hypertension and CVD. Table 1 details the weighted baseline characteristics categorized by BPH status.

**Table 1:** Weighted characteristics of participants according to BPH classification.

Characteristics	Total (*n* = 2,374)	Benign prostatic hyperplasia		*p* value
		No (*n* = 1931)	Yes (*n* = 443)	
Age (year), (mean ± SD)	56.7 ± 11.6	55.2 ± 11.1	63.9 ± 10.9	<0.001^*^
Caffeine (100 mg/d), (mean ± SD)	2.31 ± 2.31	2.35 ± 2.40	2.13 ± 1.85	0.8
Race, *n* (%)				
Non-Hispanic White and Non-Hispanic Black	1,829 (88%)	1,451 (87%)	378 (92.1%)	0.054
Mexican American	342 (5.7%)	305 (6.0%)	37 (4.3%)	
Other Hispanic	141 (2.6%)	118 (2.8%)	23 (1.9%)	
Other races - including Multi-Racial	62 (3.7%)	57 (4.2%)	5 (1.7%)	
Educational level, *n* (%)				
<High school	346 (7.0%)	296 (7.2%)	50 (5.9%)	0.4
≥High school	2,028 (93%)	1,635 (92.8%)	393 (94.1%)	
PIR, *n* (%)				
< 1.3	552 (14%)	476 (15%)	76 (9.0%)	0.066
1.3–3.5	906 (33%)	725 (32%)	181 (37%)	
≥3.5	916 (53%)	730 (53%)	186 (54%)	
Smoking status, *n* (%)				
Nonsmoker	896 (41%)	758 (42%)	138 (35%)	<0.001^*^
Former smoker	988 (38%)	735 (35%)	253 (53%)	
Current smoker	490 (21%)	438 (23%)	52 (12%)	
Marital status, *n* (%)				
Married/living with partner	2,109 (88%)	1,693 (88%)	416 (93%)	0.058
Live alone	265 (12%)	238 (12%)	27 (7.0%)	
BMI status, *n* (%)				
BMI < 18.5	514 (20.1%)	408 (19.8%)	106 (22.2%)	0.6
18.5 < BMI < 25	27 (0.8%)	22 (0.4%)	5 (0.6%)	
BMI ≥ 25	1,833 (79.1%)	1,501 (79.8%)	332 (77.2%)	
Uric acid (mg/dl)				
<5.6	780 (31%)	625 (29%)	155 (38%)	0.12
5.5–6.6	795 (35%)	652 (36%)	143 (31%)	
≥6.6	799 (34%)	654 (35%)	145 (31%)	
Diabetes, *n* (%)				
No	1,816 (82%)	1,494 (83.4%)	322 (78%)	0.069
Yes	558 (18%)	437 (16.6%)	121 (22%)	
Hypertension, *n* (%)				
No	1,065 (50%)	914 (53%)	151 (38.5%)	<0.001^*^
Yes	1,309 (50%)	1,017 (47%)	292 (61.5%)	
Hyperlipemia, *n* (%)				
No	739 (29%)	609 (29%)	130 (29%)	0.9
Yes	1,635 (71%)	1,322 (71%)	313 (71%)	
Alcohol consumption, *n* (%)				
Nondrinker or Light drinker	1,913 (78%)	1,535 (77%)	378 (80%)	0.3
Heavy drinker	461 (22%)	396 (23%)	65 (20%)	
History of CVD, *n* (%)				
No	2,135 (91.8%)	1,773 (93.3%)	362 (83.5%)	<0.001^*^
Yes	239 (8.2%)	158 (6.7%)	81 (16.5%)	

SD, standard deviation; PIR, poverty income ratio; CVD, cardiovascular disease; BMI, body mass index; ^*^p < 0.05.

Baseline characteristics are described according to tertiles of caffeine intake in Table 2. Participants were divided into T1 (*n* = 1,018), T2 (*n* = 773), and T3 (*n* = 583) according to tertiles of caffeine intake. Age, race, educational level, PIR, smoking status, uric acid, diabetes, and hypertension varied significantly among the three groups (*p* < 0.05).

**Table 2:** Weighted characteristics of participants according to caffeine intake classification.

Characteristics	Total (*n* = 2,374)	Caffeine intake			*P* value
		Tertile 1 (<106.5 mg)	Tertile 2 (106.5–261.5 mg)	Tertile 3 (≥261.5 mg)	
Age (year), (mean ± SD)	56.7 ± 11.6	58.5 ± 12.7	57.1 ± 11.6	54.6 ± 10.0	<0.001^*^
Race, *n* (%)					
Non-Hispanic White and Non-Hispanic Black	1,829 (88%)	735 (84%)	604 (88%)	490 (92.3%)	0.014^*^
Mexican American	342 (5.7%)	187 (9.0%)	100 (5.2%)	55 (2.8%)	
Other Hispanic	141 (2.6%)	75 (3.7%)	46 (2.8%)	20 (1.3%)	
Other races - Including Multi-Racial	62 (3.7%)	21 (3.3%)	23 (4.0%)	18 (3.6%)	
Educational level, *n* (%)					
<High school	346 (7.0%)	188 (9.7%)	114 (7.5%)	44 (4.0%)	0.006^*^
≥High school	2,028 (93%)	830 (90.3%)	659 (92.5%)	539 (96%)	
PIR, *n* (%)					
< 1.3	552 (14%)	268 (17%)	172 (13%)	112 (11%)	<0.001^*^
1.3–3.5	906 (33%)	429 (40%)	276 (29%)	201 (31%)	
≥3.5	916 (53%)	321 (43%)	325 (58%)	270 (58%)	
Smoking status, *n* (%)					
Nonsmoker	896 (41%)	463 (51%)	282 (40.5%)	151 (31%)	<0.001^*^
Former smoker	988 (38%)	406 (35%)	349 (44.5%)	233 (35%)	
Current smoker	490 (21%)	149 (14%)	142 (15%)	199 (34%)	
Marital status, *n* (%)					
Married/living with partner	2,109 (88%)	893 (86%)	694 (90%)	522 (89%)	0.3
Live alone	265 (12%)	125 (14%)	79 (10%)	61 (11%)	
BMI status, *n* (%)					
BMI < 18.5	514 (20.2%)	231 (22.2%)	177 (22.1%)	106 (16.4%)	0.083
18.5 < BMI < 25	27 (0.6%)	12 (0.8%)	9 (0.9%)	6 (0.6%)	
BMI ≥ 25	1,833 (79.2%)	775 (77%)	587 (77%)	471 (83%)	
Uric acid (mg/dl)					
<5.6	780 (31%)	364 (34.5%)	244 (29%)	172 (29%)	0.048^*^
5.5–6.6	795 (35%)	308 (30.5%)	262 (34%)	225 (41%)	
≥6.6	799 (34%)	346 (35%)	267 (37%)	186 (30%)	
Diabetes, *n* (%)					
No	1,816 (82%)	754 (79.2%)	588 (82%)	474 (86.3%)	0.023^*^
Yes	558 (18%)	264 (20.8%)	185 (18%)	109 (13.7%)	
Hypertension, *n* (%)					
No	1,065 (50%)	419 (45%)	346 (48%)	300 (58%)	<0.001^*^
Yes	1,309 (50%)	599 (55%)	427 (52%)	283 (42%)	
Hyperlipemia, *n* (%)					
No	739 (29%)	306 (27%)	258 (32%)	175 (28%)	0.2
Yes	1,635 (71%)	712 (73%)	515 (68%)	408 (72%)	
Alcohol consumption, *n* (%)					
Nondrinker or Light drinker	1,913 (78%)	848 (82%)	615 (75%)	450 (76%)	0.078
Heavy drinker	461 (22%)	170 (18%)	158 (25%)	133 (24%)	
History of CVD, *n* (%)					
No	2,135 (91.5%)	907 (89.5%)	693 (92.3%)	535 (93%)	0.2
Yes	239 (8.5%)	111 (10.5%)	80 (7.7%)	48 (7%)	
BPH					
Non-BPH	1931 (82%)	836 (83%)	626 (81%)	469 (83%)	0.6
BPH	443 (18%)	182 (17%)	147 (19%)	114 (17%)	

SD, standard deviation; PIR, poverty income ratio; BPH, benign prostatic hyperplasia; CVD, cardiovascular disease; BMI, body mass index; ^*^p < 0.05.

### 3.2 Higher caffeine intake increases the risk of BPH

In the multivariable model, none of the included variables showed multicollinearity (all VIFs <2.5). Table 3 describes the relationship between caffeine intake and BPH. In Model 1, unadjusted model, caffeine intake did not show a significant association with BPH (*p* > 0.05). In Model 2, with partial adjustment for covariates, caffeine intake was associated with BPH but did not reach statistical significance (*p* = 0.057). In Model 3, adjusting for all covariates, the results indicated a positive relationship between caffeine intake and BPH, with the highest tertile of caffeine intake being associated with a 52% higher risk of BPH than the lowest tertile (OR = 1.52; 95% CI: 1.01–2.27; *p* = 0.044; *P* for trend = 0.04). Since BPH is often accompanied by LUTS, we explored the relationship between caffeine intake and LUTS. Our findings indicate that higher caffeine consumption is associated with an increased risk of LUTS, indirectly suggesting that caffeine intake may elevate the risk of BPH (Supplementary material S1).

**Table 3:** Association between caffeine intake and BPH, weighted.

Caffeine intake	Model 1		Model 2		Model 3	
	OR (95% CI)	*p* Value	OR (95% CI)	*p* Value	OR (95% CI)	*p* Value
Tertile 1	1 (Ref)		1 (Ref)		1 (Ref)	
Tertile 2	1.17 (0.84, 1.62)	0.3	1.28 (0.90, 1.81)	0.2	1.32 (0.93, 1.87)	0.1
Tertile 3	1.01 (0.69, 1.47)	>0.9	1.44 (0.99, 2.11)	0.057	1.52 (1.01, 2.27)	0.044^*^
*P* for trend	>0.9		0.053		0.04^*^	

OR, Odds ratio; 95% CI, 95% confidence interval. ^*^p < 0.05. Model 1: Unadjusted model. Model 2: Adjusted for age, race, education level, marital status, PIR, alcohol intake, smoking status and BMI status. Model 3: Model 2 and adjusted for hyperlipemia, diabetes, hypertension, uric acid and cardiovascular disease.

### 3.3 Subgroup analysis

**Figure 2:**
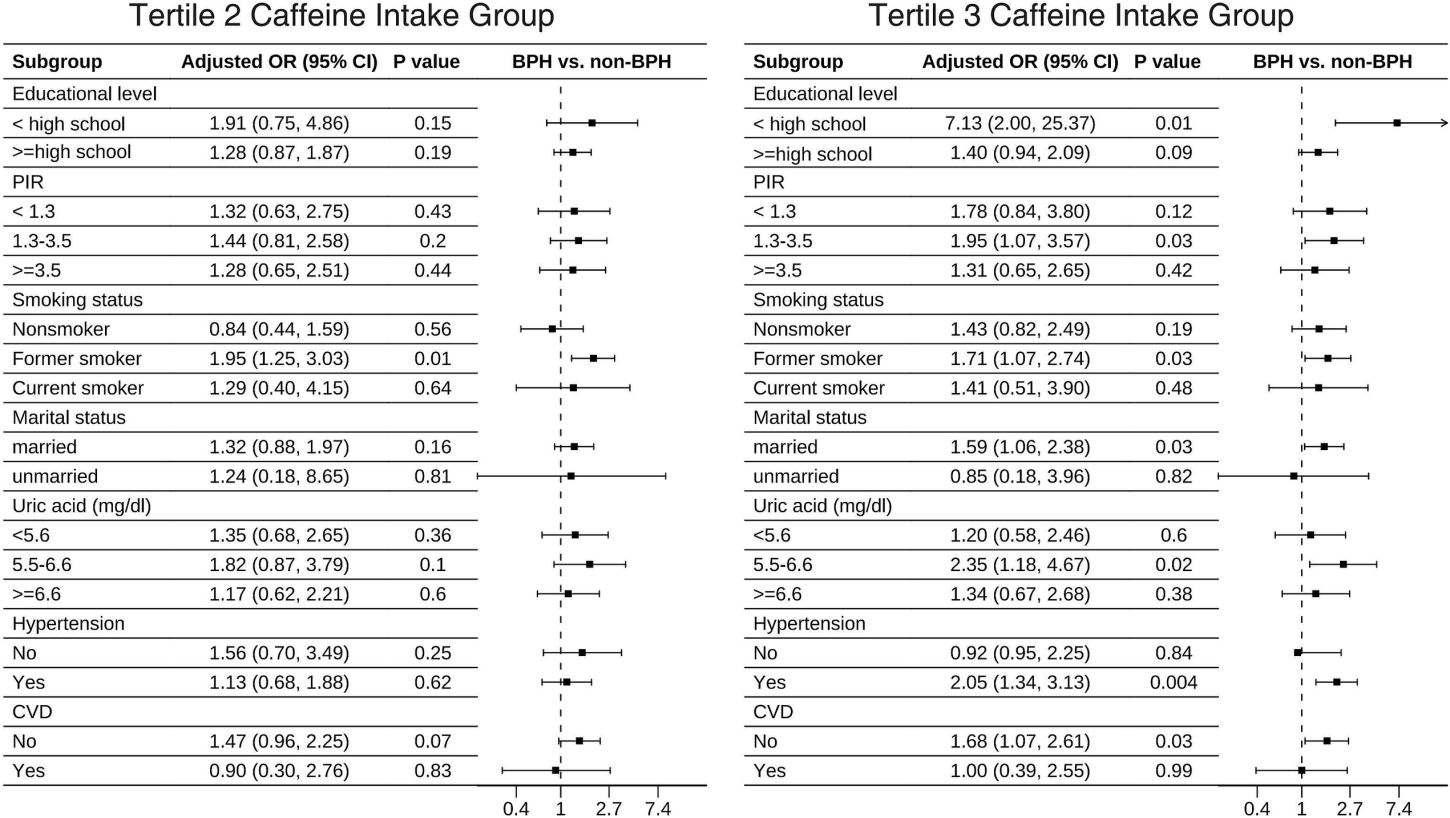
Subgroup analysis of the associations between different caffeine intake levels and BPH.

The results of the fully adjusted Model 3 subgroup analysis are displayed in Table 4. The positive association between caffeine intake and BPH remained stable when stratified by educational level, PIR, smoking status, marital status, uric acid, and the presence of hypertension and CVD. In the final forest plot (Figure 2), we found that those with lower education level (ORT_3vs1_ = 7.13, 95% CI: 2.0–25.37), a PIR of 1.3 to 3.5 (ORT_3vs1_ = 1.95, 95% CI: 1.07–3.57), former smokers (ORT_2vs1_ = 1.95, 95% CI: 1.25–3.03; ORT_3vs1_ = 1.71, 95% CI: 1.07–2.74), married/living with partner (ORT_3vs1_ = 1.59, 95% CI: 1.06–2.38), uric acid levels of 5.5–6.6 mg/dL (ORT_3vs1_ = 2.35, 95% CI: 1.18–4.67), hypertension (ORT_3vs1_ = 2.05, 95% CI: 1.34–3.13) and no CVD (ORT_3vs1_ = 1.68, 95% CI: 1.07–2.61), BPH was positively correlated with caffeine intake. We also found that there was a significant interaction effect between hypertension and caffeine intake (*P* for interaction <0.05), indicating that the promoting effect of caffeine on BPH is more significant in the hypertensive population.

**Table 4:** Weighted or (95% CI) of caffeine intake and BPH in each subgroup.

Subgroup	Tertile 1	Tertile 2	Tertile 3	*P* for trend	*P* for interaction
		OR (95% CI)	OR (95% CI)		
Educational level					
<High school	1(Ref)	1.91 (0.75, 4.86)	7.13 (2, 25.37) ^*^	0.003 ^*^	0.05
≥ High school	1(Ref)	1.28 (0.87, 1.87)	1.4 (0.94, 2.09)	0.09	
PIR					
< 1.3	1(Ref)	1.32 (0.63, 2.75)	1.78 (0.84, 3.8)	0.11	0.59
1.3–3.5	1(Ref)	1.44 (0.81, 2.58)	1.95 (1.07, 3.57) ^*^	0.03 ^*^	
≥3.5	1(Ref)	1.28 (0.65, 2.51)	1.31 (0.65, 2.65)	0.42	
Smoking status					
Nonsmoker	1(Ref)	0.84 (0.44, 1.59)	1.43 (0.82, 2.49)	0.27	0.08
Former smoker	1(Ref)	1.95 (1.25, 3.03)	1.71 (1.07, 2.74) ^*^	0.03 ^*^	
Current smoker	1(Ref)	1.29 (0.4, 4.15)	1.41 (0.51, 3.9)	0.52	
Marital status					
Married/living with partner	1(Ref)	1.32 (0.88, 1.97)	1.59 (1.06, 2.38) ^*^	0.03 ^*^	0.35
Live alone	1(Ref)	1.24 (0.18, 8.65)	0.85 (0.18, 3.96)	0.88	
Uric acid (mg/dL)					
<5.6	1(Ref)	1.35 (0.68, 2.65)	1.2 (0.58, 2.46)	0.55	0.8
5.5–6.6	1(Ref)	1.82 (0.87, 3.79)	2.35 (1.18, 4.67) ^*^	0.02 ^*^	
≥6.6	1(Ref)	1.17 (0.62, 2.21)	1.34 (0.67, 2.68)	0.36	
Hypertension					
No	1(Ref)	1.56 (0.7, 3.49)	0.92 (0.38, 2.25)	0.76	0.01^*^
Yes	1(Ref)	1.13 (0.68, 1.88)	2.05 (1.34, 3.13) ^*^	0.004 ^*^	
History of CVD					
No	1(Ref)	1.47 (0.96, 2.25)	1.68 (1.07, 2.61) ^*^	0.02 ^*^	0.43
Yes	1(Ref)	0.9 (0.3, 2.76)	1 (0.39, 2.55)	0.97	

CVD, cardiovascular disease; PIR, poverty to income ratio; CI, confidence interval. Adjusted for age, race, education level, marital status, PIR, alcohol intake, smoking status, BMI, hyperlipemia, diabetes mellitus, uric acid, hypertension, and cardiovascular disease. ^*^p < 0.05.

## 4 Discussion

This study, which revealed a positive correlation between caffeine intake and BPH, is the first to focus on the relationship between caffeine intake and BPH. From the NHANES database, we used a comprehensive, nationally representative sample of 2,374 participants, and after adjusting for a range of potential confounders, we found that participants with higher caffeine intake was associated with an increased risk of developing BPH.

Caffeine is very common in the global diet (35), and numerous studies have shown that caffeine is associated with a reduced risk of depression, Parkinson’s disease, liver disease, and cardiovascular mortality, among others (13–16). However, the relationship between caffeine intake and BPH remains controversial (22, 36–38). In our study, we found that caffeine intake was positively associated with BPH. Interestingly, subgroup analysis revealed a significant association between caffeine intake and BPH in subgroups with lower education levels, a PIR between 1.3 and 3.5, former smokers, married/living with partner individuals, individuals with uric acid levels between 5.5 and 6.6 mg/dL, those with hypertension, and those without CVD. For instance, in the T3 caffeine intake group, participants with lower education levels, hypertension, and no CVD had a higher prevalence of BPH. This may be puzzling. Here, we provide a plausible explanation: individuals with lower education levels may not pay attention to healthy lifestyles and habits, and choose more high-caffeine drinks, such as sugary drinks and energy drinks; hypertensive patients’ blood vessels already endure greater pressure, and long-term caffeine intake may lead to sustained vasoconstriction, potentially affecting blood supply to the prostate and resulting in abnormal prostate tissue growth; for participants with CVD, the lack of a significant association could be due to the fact that they were taking relevant therapeutic drugs, which led to a bias in the results.

*In vitro* and animal studies, caffeine has been shown to have biological effects that may affect prostate epithelial cell proliferation. Wu et al. (39) found that caffeine can induce Ca2+ transients in primary prostate stromal cells, which may be associated with the regulation of prostate stromal cell proliferation. Other experimental animal studies have indicated that caffeine raises plasma testosterone levels by stimulating testicular interstitial cells sympathetically, but it also leads to testicular atrophy and impaired spermatogenesis. Moreover, caffeine may promote androgen-dependent prostate hyperplasia by interfering with androgen circulation (40, 41). Sarobo et al. (4) reported that caffeine intake increased plasma testosterone and dihydrotestosterone levels in Wistar rats and promoted the proliferation of epithelial cells and enhanced androgen receptor expression in the ventral prostatic lobe. Additionally, it has been proposed that caffeine may contribute to BPH through the stimulation of the sympathetic nervous system (42). Consistent with this hypothesis, Huang et al. (43) reported that chronic stress-induced sympathetic overactivity led to hyperplasia in the ventral lobe of Wistar rats, with the dorsolateral lobe mostly unaffected, and no hyperplasia was observed after chemical sympathectomy during stress. Another study on Sprague–Dawley rats revealed that unilateral sympathectomy reduced the ventral prostate weight and DNA and protein content on the lesioned side, while unilateral parasympathectomy increased these parameters on the intact side (44). Based on the above findings, we speculate that caffeine may promote BPH by stimulating the sympathetic nervous system and modulating androgen signaling pathways.

The etiology of BPH is complex and diverse, involving multiple factors such as genetics, hormones, inflammation and lifestyle. It has been suggested that caffeine may contribute to the development and progression of BPH through interactions with these factors. Caffeine metabolism is significantly influenced by genetic factors, particularly in association with CYP1A2 gene polymorphisms (45). These genetic differences may lead to individual differences in the rate of caffeine metabolism and thus influence the physiological status of the prostate gland. Androgens play a critical role in the pathogenesis of BPH, previous studies have found that caffeine can increase plasma testosterone levels by stimulating sympathetic activity in Leydig cells (40, 41), which may promote androgen-dependent BPH. In addition, inflammation is considered to be one of the important pathological mechanisms of BPH (46). Caffeine has been shown to modulate the balance of proinflammatory and anti-inflammatory factors *in vivo*, such as the concentrations of interleukin 6 and interleukin 10 (47), which may alter the inflammatory microenvironment of the prostate and thus further participate in the development of BPH. Additionally, caffeine impacts lifestyle-related metabolic diseases, such as obesity and diabetes, which are important risk factors for BPH by regulating metabolic activity or fluid balance (48–50). Previous studies have suggested that sleep disorders are risk factors for BPH (51, 52), and given caffeine’s well-documented effects on sleep quality, its role in BPH may be partly mediated through these sleep disturbances.

Caffeine, as a bioactive compound, has a variety of pharmacological effects, which can promote sympathetic activation leading to smooth muscle contraction, which in turn promotes BPH, and sympathetic activation can also modulate testosterone levels and prostatic blood supply (4, 42), and these factors play an important role in the pathogenesis of BPH (53). In addition, caffeine may exacerbate BPH by altering local inflammatory status and oxidative stress levels in the prostate. These properties may make the effect of caffeine on BPH different from other dietary factors. In contrast, it has been shown that the association of high-calorie diets and red meat intake with BPH is mainly indirectly influenced through systemic metabolic pathways, for example, promoting the development of obesity and metabolic syndrome (6). On the other hand, vegetable may offer protective effects against BPH due to its anti-inflammatory and antioxidant properties (54).

Our study found that caffeine is more directly associated with BPH, particularly in the high caffeine intake group (T3), where the risk increased significantly (OR = 1.52, 95% CI: 1.01–2.27). In contrast, the increased risk in the medium intake group (T2) did not reach statistical significance. This suggests that caffeine may have a dose-dependent effect, with a more significant promoting effect on BPH once a certain threshold is exceeded. Future research should further clarify the safe threshold for caffeine intake and its dose–response relationship. Our subgroup analysis revealed that the association between high caffeine intake and increased BPH risk was more pronounced in specific subgroups, such as individuals with hypertension (OR = 2.05, 95% CI: 1.34–3.13). Therefore, we recommend that future BPH management guidelines explore the potential effects of caffeine intake on high-risk subgroups (e.g., those with hypertension) and assess dietary interventions based on broader research evidence. Furthermore, metabolic syndrome is well-established as an important risk factor for BPH (55, 56). Caffeine may indirectly influence the development of BPH by modulating components of metabolic syndrome. For example, caffeine may alter blood glucose levels, insulin sensitivity, and lipid metabolism (57, 58), and these metabolic changes could exacerbate obesity and chronic inflammation, thereby increasing the risk of BPH. In our study, we adjusted for key components of metabolic syndrome (such as BMI, hypertension, and diabetes), but future studies should further explore the potential mediating role of caffeine in the relationship between metabolic syndrome and BPH.

We explored the relationship between caffeine intake and BPH with a relatively large sample size extracted from the NHANES, a comprehensive, nationally representative database, which supports the generalizability of our findings among adults in the United States. In addition, we performed subgroup analyses to identify the relationship between caffeine intake and BPH across different populations. This study also has several limitations. First, as a cross-sectional study, a causal relationship between caffeine intake and BPH could not be established. To better understand the relationship between caffeine intake and BPH, future studies should include prospective designs and randomized controlled trials. Second, although we adjusted for potential confounders affecting BPH, some confounding factors may remain unconsidered, such as sleep quality and sleep duration. Third, dietary intake data were collected via 24-h dietary recalls, which may not adequately reflect long-term dietary habits. However, some studies suggest that two 24-h recalls can sufficiently assess daily dietary intake (59). Fourth, the sources of caffeine are diversified, and this study did not analyze the effects of different caffeine sources on BPH. In future research, we plan to explore the impact of various caffeine sources on BPH using more detailed data, addressing this limitation of the current study. Fifth, in this study, BPH diagnosis was based solely on questionnaire data, without objective clinical measures (such as imaging tests or laboratory markers), which may be some classification error, in future studies we will add objective clinical data on BPH to further clarify the relationship between caffeine intake and BPH.

## 5 Conclusion

Our study is the first national cross-sectional survey to explore the association between caffeine intake and BPH, and the current study provides preliminary evidence that there may be a positive association between caffeine intake and BPH. We aspire for this study to offer insights for future research into the biological mechanisms underlying the effects of caffeine on BPH.

## Data Availability

The datasets used and/or analysed during the current study are available from the corresponding author on reasonable request.
