# Supplementary material for: Exploring the association between caffeine intake and benign prostatic hyperplasia: results from the NHANES 2005–2008
Source: Front Nutr. 2025 Jan 13;11:1511607. doi: 10.3389/fnut.2024.1511607 (PMC11770993; doi:10.3389/fnut.2024.1511607)
Supplement: Supplementary file 1 [file Table_1.docx]

**Participants**

In this study, we used publicly accessible data from two 2-year cycles of NHANES (2005–2006 and 2007–2008) and restricted the analysis cohort to men aged 40 years or older. Initially, there were 7081 male participants aged 40 years and older in our data. We excluded 2079 participants with incomplete caffeine intake data or incomplete LUTS status and 77 participants with a history of prostate cancer. Finally 4925 participants were included in our study, 3195 of whom met the diagnostic criteria for LUTS.

**Questionnaire data assessment**

LUTS were assessed by three questions, including: (1) ‘Do you usually have trouble starting to urinate (pass water)?’ (hesitancy, defined as the answer is yes); (2) ‘After urinating (passing water), does your bladder feel empty?’ (incomplete emptying, defined as the answer is no); (3) ‘How often do you have urinary leakage?’ (urinary frequency, defined as the answer is 1 or greater). LUTS was defined as a participant having one or more of the mentioned symptoms [1].

**Caffeine intake assessment**

The mean caffeine intake from the two 24-hour recalls was used in the analysis, and daily caffeine intake was divided into three groups according to tertiles (T): T1 (< 86.5 mg/d), T2 (86.5-226.5 mg/d) and T3 (≥226.5 mg/d).

**Covariates**

The included variables are the same as above.

**Statistical analysis**

The included variables were tested by multicollinearity, and Variance Inflation Factor (VIF) was used to evaluate them. The association between caffeine intake and LUTS was analyzed using weighted logistic regression. Three different logistic regression models were used: Model 1 was unadjusted, Model 2 was adjusted for age, race, educational level, PIR, marital status, smoking status, alcohol consumption, and BMI. Model 3 was adjusted for all potential confounding factors, with additional adjustments for diabetes, uric acid, hypertension, hyperlipemia, and CVD in addition to those made in Model 2. All analyses were performed with R version 3.6.1, and a *P*-value of less than 0.05 was considered statistically significant.

**Result**

***Higher Caffeine Intake Increases the Risk of LUTS***

In the multivariable model, none of the included variables showed multicollinearity (all VIFs < 3). Table 1 describes the relationship between caffeine intake and LUTS. In Model 1, unadjusted model, caffeine intake shows a significant association with LUTS (*P* < 0.001). In Model 2, with partial adjustment for covariates, caffeine intake still shows a significant association with LUTS (*P* < 0.001). In Model 3, adjusting for all covariates, the results indicated a positive relationship between caffeine intake and LUTS, with the highest tertile of caffeine intake being associated with a 63% higher risk of BPH than the lowest tertile (OR = 1.63; 95% CI: 1.36-1.94; *P* < 0.001; *P* for trend< 0.001).

**Table S1: Association between caffeine intake and LUTS, weighted.**

|  | **Model 1** | | **Model 2** | | **Model 3** | |
| --- | --- | --- | --- | --- | --- | --- |
| **Caffeine intake** | **OR (95% CI)** | ***p* Value** | **OR (95% CI)** | ***p* Value** | **OR (95% CI)** | ***p* Value** |
| Tertile 1 | 1 (Ref) |  | 1 (Ref) |  | 1 (Ref) |  |
| Tertile 2 | 1.02 (0.85, 1.21) | 0.9 | 1.00 (0.82, 1.20) | ＞0.9 | 0.99 (0.82, 1.21) | ＞0.9 |
| Tertile 3 | 1.69 (1.44, 1.98) | ＜0.001^*^ | 1.61, (1.36, 1.92) | ＜0.001^*^ | 1.63 (1.36, 1.94) | ＜0.001^*^ |
| ***P* for trend** | ＜0.001^*^ |  | ＜0.001^*^ |  | ＜0.001^*^ |  |

**Abbreviations:** OR: Odds ratio; 95% CI: 95% confidence interval. ^*^*P*＜0.05.

Model 1: Unadjusted model.

Model 2: Adjusted for age, race, education level, marital status, PIR, alcohol intake, smoking status and BMI status.

Model 3: Model 2 and adjusted for hyperlipemia, diabetes, hypertension, uric acid and cardiovascular disease.

**References**

[1] Fantus RJ, Packiam VT, Wang CH, Erickson BA, Helfand BT. The Relationship between Sleep Disorders and Lower Urinary Tract Symptoms: Results from the NHANES. J Urol. 2018 Jul;200(1):161-166.
